# Real-Time Cardiac Biofeedback Intervention: Experiences of Patient and Public Involvement in a Randomized Controlled Trial

**DOI:** 10.2196/84737

**Published:** 2026-03-12

**Authors:** Marina Bobou, Michal Tanzer, Alkistis Saramandi, Caroline Selai, Paul M Jenkinson, Aikaterini Fotopoulou

**Affiliations:** 1 Research Department of Clinical, Educational and Health Psychology, University College London, 1-19 Torrington Place, London, England, WC1E 7HB, United Kingdom, 44 20 7612 6143; 2 Department of Clinical and Movement Neurosciences, UCL Queen Square Institute of Neurology, London, United Kingdom; 3 Faculty of Psychology, Counselling and Psychotherapy, The Cairnmillar Institute, Melbourne, Australia

**Keywords:** patient and public involvement, PPI, interoception, biofeedback, smartwatch, mental health, eating disorders

## Abstract

**Background:**

Patient and public involvement (PPI) is crucial for enhancing research quality and relevance and addressing health inequalities. PPI ensures that studies tackle relevant and meaningful questions, as there is a recognized need by the research community to document and share PPI studies to advance the field and encourage the adoption of such activities.

**Objective:**

The study aimed to provide a detailed report on the PPI activities undertaken to develop and implement a randomized controlled trial of a novel therapeutic tool designed to increase interoception and metacognition (ie, the Interoceptive Insight and Metacognitive Efficacy beliefs [InMe] trial).

**Methods:**

The InMe trial integrated insights from experts by experience, as well as from clinical and academic experts. This collaborative approach resulted in the development of a comprehensive trial across 4 main stages—design, delivery, results interpretation, and future planning.

**Results:**

Here, we highlighted the unique insights and the added value in incorporating PPI activities into our trial development and implementation, while reporting challenges and shortcomings that were identified throughout this process.

**Conclusions:**

PPI activities within the InMe trial led to meaningful changes, while collaborators expressed satisfaction and increased interest in interoception research. Further improvements on how to best support experts by experience when sharing their experiences were also identified.

## Introduction

### Patient and Public Involvement

Historically, research has been driven by academics and clinicians with expertise in a given field, with little or no involvement of those with lived, living, or other forms of relevant experience. Lack of involvement of individuals with relevant experience could potentially lead to a “mismatch” between the researcher and patient needs addressed, which has been described as a key contributor to research waste [1,2]. Research waste arises from poor research conduct and ambiguous reporting of research outcomes, which renders research unusable, as direct conclusions cannot be drawn [3]. Patient and public involvement (PPI) offers a potential solution to this problem. By engaging individuals with relevant experience, researchers can identify methodological drawbacks, enhance study design, improve feasibility within public health services, and ensure acceptability of interventions to patients. Additionally, PPI can ameliorate research reporting and dissemination, thereby improving the overall quality and impact of studies [4,5]. Indeed, evidence suggests that PPI initiatives provide unique perspectives, ensuring that research outcomes have meaningful impacts on service users [6-8].

According to the guidelines of the Center for Engagement and Dissemination within the National Institute for Health and Care Research (NIHR; replacing what was previously known as INVOLVE), PPI is commonly defined as research “being carried out with or by members of the public rather than to, about or for them” [9]. Here, “members of the public” refers to patients with lived or living experience, but also carers of patients directly impacted by them and health care professionals. Indeed, in the last 2 decades, there has been an increasing interest in the active involvement of patients and members of the public in research, which has become a common requirement and priority for major institutions and funders in the research innovation industry. These institutions range from the UK Medical Research Council [10] to major research charities such as the Wellcome Trust [11]. Moreover, different initiatives such as the UK Standards for Public Involvement and NIHR Center for Engagement and Dissemination have been developed to promote and guide researchers to carry out consistent and standardized PPI collaboration activities, thus allowing research to become more relevant to patients and clinicians within the health care system. Additionally, PPI initiatives have gained prominence across several European countries, with the Netherlands and Scandinavian countries being at the forefront of such developments [12,13]. Other considerations, such as the engagement and involvement of minority and marginalized communities in research, have further enhanced research equality and diversity, rendering research also relevant to underrepresented populations [14,15]. However, challenges have been observed, particularly in how members of the public are recruited and supported throughout the involvement process. These observations call for further research resources and infrastructure changes to maintain a long-term and meaningful collaboration [15].

Accordingly, the present study presents and critically evaluates the PPI activities and collaboration that occurred for the development and completion of the Interoceptive Insight and Metacognitive Efficacy beliefs (InMe) trial. In the following section, a brief summary of the randomized controlled trial’s (RCT’s) background and design will be provided, before proceeding to the aims and procedures of the PPI and concluding with the coevaluation and discussion of the advantages and disadvantages of this PPI effort.

### Intervention Background and Scope

The InMe trial was developed as part of a series of studies, aimed at developing and testing the feasibility and efficacy of a novel, behavioral therapeutic intervention that can enhance interoceptive self-efficacy beliefs, that is, the beliefs of one’s ability to regulate their internal body signals (known as interoceptive signals). Interoception is defined as the process of sensing, integrating, and interpreting physiological body signals arising from major visceral systems such as the gastrointestinal, respiratory, and cardiovascular systems [16]. Previous research has shown that dysregulations or disturbances in interoceptive processing are associated with psychopathology, including but not limited to somatic symptom disorders and eating disorders (EDs) [17-20]. Nevertheless, the evidence concerning the efficacy of interoception-based interventions remains inconclusive, indicating a need for further research and specific targeting of both physiological signals themselves and related interoceptive beliefs (for systematic review see [21]). We thus aimed to develop an intervention and first test its feasibility and efficacy in a randomized controlled setting, with a subclinical sample. Here, we report the PPI activities that informed the development of the InMe trial, following its efficacy testing in a subclinical RCT, which is fully reported elsewhere [22].

### Overview of Trial Procedures

**Figure 1.**
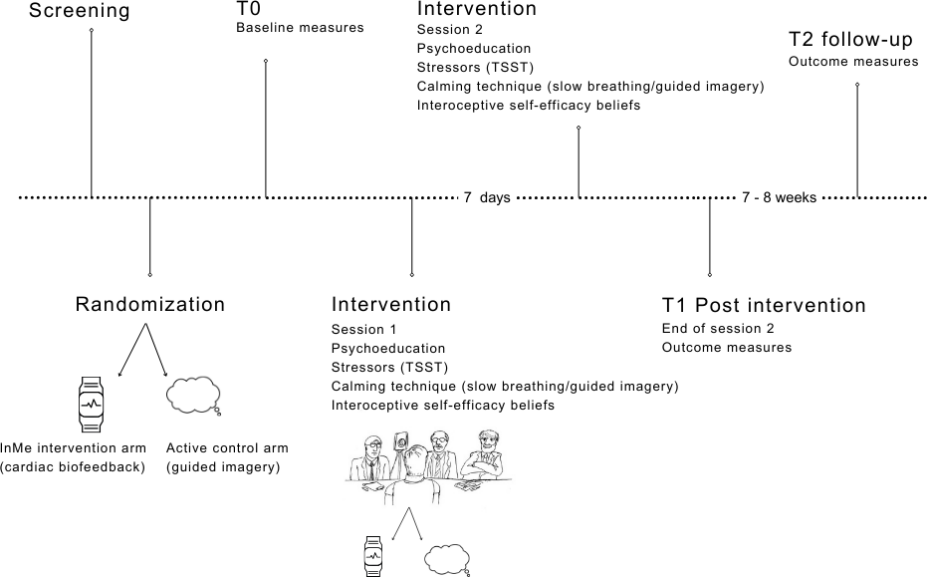
Interoceptive Insight and Metacognitive Efficacy beliefs trial timeline and procedures. TSST: Trier Social Stress Test.

The subclinical trial aimed to investigate whether the InMe intervention, which incorporated slow breathing, cardiac biofeedback, and interoceptive beliefs training, could significantly enhance interoceptive self-efficacy beliefs under stressful conditions, in comparison to a control intervention. The trial recruited a general population, predominantly university students, with low interoception as measured by the Body Awareness Questionnaire [23]. Participants were randomized into 2 groups: the InMe intervention group or an active control group that used a guided imagery technique. After randomization, participants completed a comprehensive set of baseline questionnaires, designed to measure symptoms including disordered eating, as measured by the Eating Disorder Examination Questionnaire [24], and somatic symptom disorders measured by the Patient Health Questionnaire-15 [25]. As part of the intervention, participants were provided with psychoeducation about heart rate, including how heart rate fluctuates under stress and can be regulated. Following this educational component, participants underwent a stress induction procedure using the standardized Trier Social Stress Test (TSST) [26]. During the TSST, participants were instructed to use either the slow breathing technique (InMe intervention) or the guided imagery technique (active control) to downregulate their stress response and build related self-efficacy beliefs. Seven days after the initial session, participants repeated the intervention and completed postintervention measures. Finally, a follow-up session was conducted 7‐8 weeks after the intervention to evaluate sustained effects. The detailed InMe trial procedures are shown in Figure 1.

### Aims of the PPI

The aim of this paper was to provide a detailed report on the involvement and contribution of patients and members of the public in the development of the InMe trial. PPI has been a key element in the conception, design, development, and completion of the InMe trial, with the involvement of external collaborators also playing a significant role in the interpretation of trial results and informing future clinical trial planning and practice. Additionally, this paper aims to provide insights regarding challenges that arose during each phase of the research process and offers suggestions on how research teams incorporating PPI might address similar challenges in the future. An overview of the PPI activities and the aims achieved within the framework of this collaboration is presented below (Table 1).

**Table 1.** Detailed list of the PPI^a^ aims and time frames within which they should be achieved.

Aims	PPI activities				
	Discussion 1	Email	In person	Discussion 2	Discussion 3
Development of operating procedures	✓				
Outcome measure development	✓				
Sample specific stressor	✓				
Statistical analysis plan development		✓			
Development of participant-facing documents		✓			
Trial advertisement strategy		✓			
Research team training & optimization			✓		
Optimization of intervention procedures			✓	✓	
Consideration of development of patient-specific stressors				✓	
Discussion on treatment differences in EDs^b^ and somatic symptom disorders				✓	
Planning for implementation in a clinical setting				✓	
Adherence to intervention					✓
Considerations on breathing exercise and biofeedback					✓
Discussion on use of technology barriers within the NHS^c^					✓
Further consideration on patient-specific trial procedures and stressors					✓

a PPI: patient and public involvement.

b EDs: eating disorders.

c NHS: National Health Service.

## Methods

### Collaboration Approach

Email invitations were sent to experts by experience (of EDs and/or somatic symptom disorders), health care practitioners, and researchers, to collaborate on the development of the InMe trial. These individuals’ contact details were obtained from previous clinical and social media contacts. Meetings took place online due to location and time constraints; collaboration occurred in the form of online group discussions, online focus groups, and email communications, supported by audio recordings and note taking. The InMe trial was funded by a small grant from InMe, United Kingdom. Within this grant, there was no budget available for the reimbursement of PPI activities for contributors. Therefore, participation in all PPI activities was voluntary, with full administrative support provided by the research team. Engagement expectations of PPI contributors regarding their role in helping to develop and co-design trial procedures were discussed and formalized beforehand via email.

### Ethical Considerations

Institutional ethics approval was granted in February 2022 for the InMe trial, including all its feasibility and other participant feedback measures, under the University College London ethical amendment (CEHP/2019/577; Body to Mind Awareness). Guidance from government organizations (eg, UK Health Research Authority) and recent academic, peer-reviewed work [27] suggests that activities such as the present PPI work do not require formal ethical approval from an ethics committee. All participants were informed about the collaboration, their role and involvement, the conditions of anonymity and confidentiality, and agreed to be involved. InMe trial participants received written information about the study’s objectives, procedures, and potential risks before providing written informed consent. Those who participated in the RCT were offered a choice of either £37 (US $49.50) or credit points (1 credit point per hour of participation, applicable to University College London psychology students only) as reimbursement upon completing the study.

### Collaborators

The lived experience advisory panel members (also referred to as “experts by experience”) were selected based on their self-declared experience with somatic symptom disorders or EDs. Specifically, the lived experience advisory panel (n=5) was comprised of members with functional neurological disorder (n=1), eating disorder diagnoses (EDs; n=3), and other diagnoses (n=1). The clinical-academic steering committee members (n=10) included UK Health and Care Professions Council-registered clinical and counseling psychologists (n=3), a consultant neurologist and a senior academic neurological psychotherapist working within the National Health Service (NHS) (n=2), academics conducting research relevant to the trial (n=3), and statisticians advising on trial methodology (n=2).

### PPI Process

**Figure 2.**
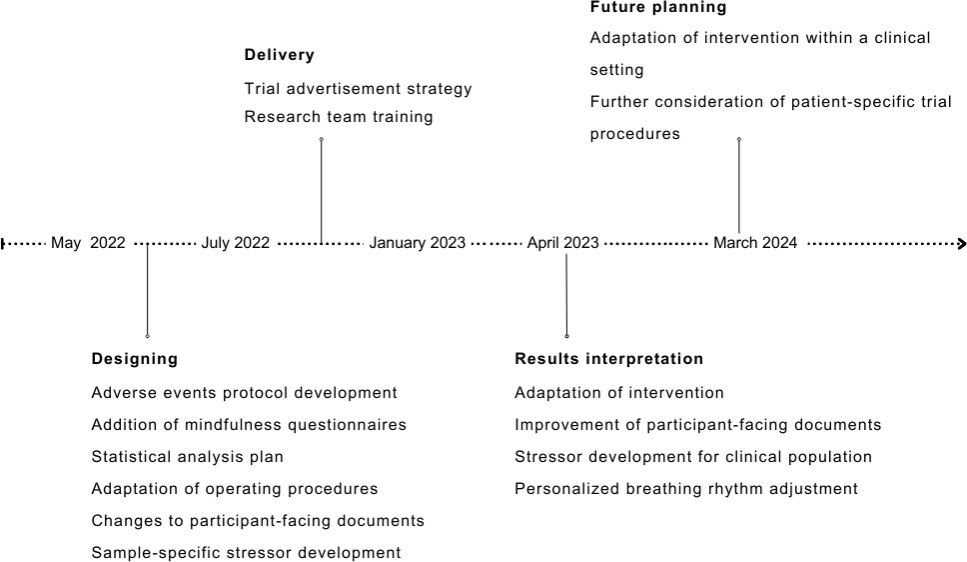
Designing, delivery, results interpretation, and future planning stages of the Interoceptive Insight and Metacognitive Efficacy belief trial.

Initial planning of the current RCT procedures was informed by the findings of an audit study conducted by the core research team, which also involved PPI collaborations with clinicians and patients within an ED ward in an NHS facility [28]. In their clinical audit, the authors aimed to understand and evaluate whether cardiac biofeedback practice following stress-inducing ward activities (here, weekly meal-planning) was better than existing, local standards of care for anorexia nervosa in helping individuals downregulate their heart rate and increase their self-efficacy beliefs about such abilities [28]. Based on the knowledge and experience gained during this audit about the potential of using biofeedback and interoceptive self-efficacy belief training to enhance existing standard practice, we aimed to develop and undertake a subclinical study testing the role of the InMe biofeedback intervention in collaboration with experts by experience, as summarized in Figure 2.

Lived experience advisory panel and clinical-academic steering committee collaborators were invited to 3 online group discussions in May 2022, April 2023, and March 2024. During the first discussion, researchers introduced and presented the preliminary suggestions for the trial design, which led to the development of the participant-facing documents. Additionally, the research team conducted a distinct discussion group to facilitate the development of the statistical analysis plan with members of the clinical-academic steering committee. Each online discussion was recorded and transcribed into text, which facilitated the research team in identifying key discussion points and feedback provided by the collaborator groups. Following the discussion, collaborators received a lay summary of the meeting including all key points and how these could be addressed by the research team. Near the completion of the trial, during the second discussion, preliminary data were presented, and collaborators discussed and reflected on the feasibility of the RCT in a broader health care setting. Following data analysis, collaborators were invited to a final discussion, in which the research team presented the main analysis results and additional feasibility findings, along with participants’ feedback on the trial procedures.

### Evaluation and Measurement of PPI Impact

PPI outcomes and impacts were qualitatively explored using an impact log, in which all email communications and online meetings were recorded. The impact log was developed according to the Public Involvement Impact Assessment Framework, which provides guidelines on how researchers can measure the impact of participant involvement [29,30]. The Public Involvement Impact Assessment Framework informed the development of the research log by first considering the study’s context and design and then developing a more tailored PPI approach to achieve the desired outcomes and was considered the most appropriate for the aims of the present PPI [31]. Moreover, it highlighted the importance of identifying barriers and facilitators in the process to ensure a successful and productive PPI collaboration. Following 2 online PPI group discussions in April 2023 and March 2024, collaborators completed an anonymized feedback form to identify future challenges and shortcomings not previously recognized or identified by the research team. Participants rated satisfaction on a 10-point Likert scale, with 0 being “not at all satisfied” and 10 being “Extremely satisfied.” Additionally, collaborators were asked an open-ended question on their overall experience participating in this trial.

## Results

### Overview

The research team worked together with the lived experience advisory panel and clinical-academic steering committee members to generate a comprehensive trial protocol and procedures, which are all presented and discussed in four stages below: designing, research delivery, results interpretation, and future planning. All procedures have been reported in accordance with the Guidance for Reporting Involvement of Patients and the Public, Version 2 reporting guidelines for PPI in research [32], the impact of which is summarized in Table 1.

### Design

Over 4 months, the research team developed a comprehensive research protocol and standardized operating procedures (SOPs) aligned with the trial’s aims. At this stage of the design process, a group discussion meeting was convened to gather clinical expertise from health care professionals and insights from patients on the intervention. During the online discussion held in May 2022, researchers presented the intervention’s objectives, trial measurements, and an overview of the procedures. Participants were encouraged to provide feedback during the presentation, followed by an open discussion. This approach was widely regarded by the research team as a key facilitator for fostering dynamic and productive conversations [33]. The discussion was centered on two primary points: (1) individuals’ experience with slow breathing as a calming technique and (2) individuals’ responses under stressful conditions. A lived experience advisory panel member noted that slow-paced breathing might pose challenges for individuals without prior experience. This feedback led to the development of an adverse event protocol to guide researchers in responding to issues such as difficulty breathing, light-headedness, or panic attacks during the slow breathing exercise or stressors. The development of this protocol underscored the importance of participant safety and demonstrated how input from experts by experience could enhance the trial’s value and procedures. Additionally, to better understand variability in breathing practices, the research team incorporated baseline questionnaires to assess participants’ previous experience with slow breathing and other mindfulness or breathwork techniques. Following consultation with a clinical-academic steering committee member, the TSST, which involves mental arithmetic and free speech in front of experimenters, was modified to better suit a sample of 18- to 30-year-old participants, predominantly university students. The adapted stressor procedures were refined and summarized in a document circulated via email to all attendees, who were invited to provide further comments on the implemented changes.

In May 2022, an in-person meeting was arranged for a clinical-academic steering committee and a lived experience advisory panel member to evaluate and optimize the trial’s assessment procedures. This resulted in improvements to the language of the SOP, adjustments to TSST timing, and reorganization of the panel of experimenters. Due to scheduling challenges, the members attended separately. After refining the stressor description and duration, the research team consulted directly with a clinical-academic steering committee member experienced in conducting TSSTs, who validated the changes to the stressor procedures.

Email communications also had a pivotal role during the design stage, facilitating the refinement of participant-facing documents such as consent forms, information sheets, and SOPs for researchers. A collaborator with expertise in research methods and statistics contributed via email to the development of a descriptive statistical analysis plan. Additionally, psychoeducation procedures, where participants were informed about fluctuations in their heart rate and breathing rhythm, were reviewed by a clinical-academic steering committee member. The psychoeducation materials for both the intervention and active control arms were optimized to ensure consistent structure and language, allowing for a direct comparison between groups. Email communication yielded impactful changes, as outlined in Table 2 below.

**Table 2.** Impact log for all patient and public involvement activities within the InMe^a^ trial.

Stage and date (MM/YY)		Involvement task	Collaborator	Outcome	Impact
Designing					
	05/22	Group discussion	Lived experience advisory panel member	Discussion on slow breathing challenges	Development of a separate adverse event protocol in the occasion of an adverse event, for participant safety
	05/22	Group discussion	Clinical-academic steering committee member	Discussion on participant’s prior and current experience with mindfulness, yoga, deep breathing, related apps, and self-tracking technologies	Added prior experience questions to baseline battery of questionnaires prior and after intervention
	05/22	Group discussion	Clinical-academic steering committee member	Discussion on how stressors can variably affect clinical populations	Development of a separate adverse event protocol to ensure participant safety
	05/22	In-person meeting	Clinical-academic steering committee member	Feedback on psychoeducation by English language native speaker	Adjusted SOP^b^ text
	05/22	In-person meeting	Lived experience advisory panel member	Reviewing TSST^c^ components and timing	Adjusted SOP and stressor length
	05/22	Email	Clinical-academic steering committee member	Development of statistical analysis plan	Adjustments on design protocol before trial preregistration
	06/22	Email	Clinical-academic steering committee member	Discussion on TSST for the intervention	Adapting TSST to fit the purposes of the intervention
	07/22	Email	Lived experience advisory panel member	Reviewing consent form and participant information sheet	Clarifying the right of participant withdrawal in consent form and separately added to SOP
	07/22	Email	Lived experience advisory panel member	Reviewing consent form and participant information sheet	No adaptation, as lived experience advisory panel considered the documents clear and concise
	07/22	Email	Clinical-academic steering committee member	Comparison of control arm guided imagery vs psychoeducation and slow breathing practice texts	Added psychoeducation component to active control arm to allow comparison
Delivery					
	07/22	Email	Lived experience advisory panel member	Provided key points to be included in the advertisement	Designing the trial advertisement
	07/22	Email	Clinical-academic steering committee member	Discussion on target population to recruit and advertisement adaptation	Produced 2 separate advertisements to be published on different media platforms
	07/22	Email	Lived experience advisory panel member	Advertisement dissemination	Redesigning the advertisement, removed barcode and cut down on text
Results interpretation					
	04/23	Group discussion	Clinical-academic steering committee member	Patients with EDs^d^ and somatic symptoms are less likely to be interested in the intervention	Researchers should opt for a graded exposure technique into the trial.
	04/23	Group discussion	Lived experience advisory panel member	For patients with somatic symptom disorders, accessibility to research site should be considered.	Information sheet should provide all the information including maps and accessibility, prior to the participant’s visit.
	04/23	Group discussion	Clinical-academic steering committee member	Generate a diagnosis-specific stressor	Meal planning for EDs and push test for somatic symptom disorders. For both describing their worst day in terms of symptoms
	04/23	Group discussion	Clinical-academic steering committee member	Not at all participants reacted the same to slow breathing to reduce stress	Aim to tailor breathing rhythm to each participant
	04/23	Group discussion	Lived experience advisory panel member	Engagement of results and praise for future research	Agreement with discussion
Future planning					
	03/24	Group discussion	Clinical-academic steering committee member	InMe module could be used as prehabilitation or add-on to standard of care.	Consideration on prehabilitation & standard treatments the module could be paired with
	03/24	Group discussion	Lived experience advisory panel member	Prehabilitation could be challenging in EDs	Focusing only on standard of care as participants would lack further support in prehabilitation
	03/24	Group discussion	Clinical-academic steering committee member	Discussion on how EDs and somatic symptom disorder populations respond to treatment	Consideration on application of the InMe module in somatic symptom disorders

a InMe: Interoceptive Insight and Metacognitive Efficacy belief.

b SOP: standardized operating procedures.

c TSST: Trier Social Stress Test.

d ED: eating disorder.

### Research Delivery: Outcomes and Impact

**Figure 3.**
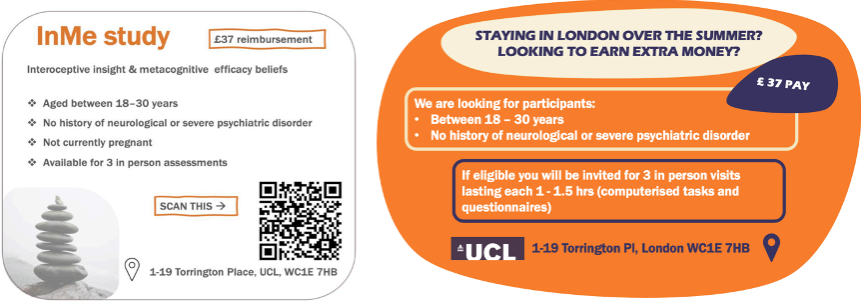
Advertisement before and after consultation with lived experience advisory panel members.

Following the formalization and approval of trial procedures, the research team proceeded to plan the participant recruitment strategy. At this stage, after being provided with guidelines for the advertisement, a lived experience advisory panel member generated the InMe trial poster, targeting individuals aged between 18‐30 years. Furthermore, with additional feedback from a clinical-academic steering committee advisor and another lived experience advisory panel member, the team improved the poster layout and content, which is visually presented in Figure 3.

### Results Interpretation

#### Outcomes and Impact

Once the preliminary analysis was conducted, the research team invited the lived experience advisory panel and clinical-academic steering committee collaborators to an online 1.5-hour discussion in April 2023. During this meeting, the efficacy and feasibility outcomes relevant to the stressors and participant acceptability of the procedures were presented. Presentation of the RCT outcomes on efficacy and feasibility goes beyond the scope of this paper and is presented fully elsewhere [22]; however, results will be briefly outlined here to contextualize the relevant PPI activities. The online discussion in April 2023 was centered on the main trial results, their clinical impact, and exploring ways to further optimize the intervention. During the discussion, two points were raised: (1) where the InMe trial intervention could be used and whether it should be used in conjunction with standardized treatments and (2) whether patients with somatic symptom disorders and EDs should be treated similarly or whether clinical differences should be considered.

#### Trial Outcomes

Results indicated that the InMe intervention was efficacious in improving the primary outcome of the RCT, namely interoceptive awareness, as measured by the Multidimensional Assessment of Interoceptive Awareness [34]. Improvements were noted in both trial arms immediately postintervention, but only participants in the InMe arm—and not those in the active control arm—experienced a maintained improvement at follow-up, which occurred 2 months postintervention. Attendees expressed an interest in the results but highlighted that patients with EDs and somatic symptom disorders might hesitate to participate in an intervention involving stressors. Additionally, considerations regarding accessibility to the study site were discussed by a lived experience advisory panel member, especially in cases of mobility challenges. From experience, experts with somatic symptom disorders emphasized that all information about accessibility to the study site should be provided in the information sheet. Thus, coproduction of participant-facing documents in future trials would be especially beneficial as it would help streamline such challenges. Regarding feasibility outcomes, the trial was deemed feasible and acceptable by participants, as TSST procedures were shown to effectively increase participants’ heart rates, and similarly, the slow breathing technique effectively reduced their heart rates.

#### Intervention and Standardized Treatment

Following the results interpretation, as the InMe intervention was deemed feasible, a clinical-academic steering committee member with clinical experience suggested that going forward, the InMe intervention could be paired with an established standard of practice therapy. The practice of the InMe intervention can be monitored and encouraged differently in each setting. During the discussion, the possibility of digitizing the InMe intervention for home practice was proposed by a researcher. However, it was concluded that participants awaiting treatment might lose motivation to participate in a self-paced intervention. In addition, a lived experience advisory panel member noted that the intervention should not be carried out online, as it might induce anxiety since some individuals with EDs disregard bodily sensations as a coping mechanism [35]. The differing perspectives between researchers and lived experience experts regarding the future use of the trial prompted further constructive dialog within the research team. A clinical-academic steering committee member suggested the intervention be used as prehabilitation to support individuals on long waiting lists for standardized treatment. This proposal was mutually agreed upon, as it would help ensure early patient engagement with the relatively easy-to-use technique, potentially offering a temporary solution to patients on waiting lists before standard treatment becomes available.

#### Treatment Differences Among EDs and Somatic Symptom Disorders

On the second discussion point, as results indicated that no other symptoms apart from disordered eating symptoms improved following the intervention, a lived experience advisory panel attendee raised a question about whether ED and somatic symptom disorder patients should be treated similarly when planning future research. The InMe intervention showed clear improvements in EDs but not in somatic symptom disorders, signifying further ambiguity in the latter. It was discussed that, in both cases, participants experience bodily uncertainty, and an intervention such as the one presented should be beneficial if administered appropriately. However, the mechanisms maintaining symptoms in EDs and somatic symptom disorders may rely on processes beyond those targeted by the presented InMe intervention, and these mechanisms may vary between populations.

### Future Planning

#### Outcomes and Impact

After the completion of the trial, the research team invited members of the clinical-academic steering committee and lived experience advisory panel for an evidence-based discussion on conducting the intervention within a health care setting. The discussion occurred in March 2024 and lasted 2 hours in total. The discussion aimed to understand if the proposed therapeutic biofeedback intervention could be translated into a clinical setting, and how it should be adjusted moving forward. Additionally, during the discussion, attendees were presented with the recruitment and dropout rates to enable further discussion. The three points discussed were the following: (1) Adherence to intervention in EDs and somatic symptom disorder populations, (2) The nature of stressors according to the patient’s clinical profile, and (3) Biofeedback, slow breathing, and how this can be used in a clinical setting.

#### Adherence to Intervention

From recruitment outcomes, a researcher (MT) responsible for participant randomization and stratification observed that participants with disordered eating behaviors and somatic symptoms were more likely to withdraw from the trial, even prior to attending the first session. Overall, higher drop-out rates have been observed in outpatient facilities compared to in-patient, as patients might experience a drop in motivation. Additionally, a barrier identified from the lived experience advisory panel, which can complicate adherence in terms of the intervention (considering that the aim of the intervention was to increase self-efficacy by providing explicit biofeedback), was the fear of patients with EDs attending to their bodies [36]. Expanding on these barriers, a clinical-academic steering committee member with expertise working with ED populations suggested that moving forward, a more graded exposure to the cardiac biofeedback intervention would be appropriate, rather than an intensive 2-session intervention. Such observations are considered useful as they will aid in developing an intervention, which will evoke interest in research participants and result in higher adherence rates, following consideration of the previous suggestions made.

#### Stressor Future Considerations

Another point on the intervention brought into discussion was the nature of the stressors. The InMe trial stressors were based on the TSST protocol, changing the free speech stressor to a stressor more relevant to a student sample (ie, an interview to join a prestigious university society). However, in the group discussion, clinical-academic steering committee members considered that future studies should develop clinically relevant stressors based on the participant’s diagnosis. For instance, in the case of EDs, participants could potentially be asked to plan their meals for the weekly schedule (as in our initial clinical audit [28]), which is stress-inducing but also clinically relevant. Further, in the case of somatic symptom disorders, a clinical-academic steering committee member suggested that participants could be invited to explain their worst day in terms of symptoms, perform the Hoover sign or tremor entrainment test [37], which is also considered stressful and triggering to patients. Both suggestions will be piloted by the research team in future clinical work, investigating if stressor relevance to diagnosis could be effective in evoking a stress response, and thus increasing participants’ heart rate.

#### Biofeedback Future Considerations

As a last discussion point, the research team suggested the possibility of the slow breathing rhythm being adjusted to each participant, rather than all participants following the standardized slow breathing technique [38]. Effectively, adjusting the breathing technique to the individual’s breathing rhythm has optimal effects in improving mood and relaxation, while reducing the possibility of adverse events [39]. However, implementing a tailored breathing rhythm raises concerns regarding technical complexity and scalability. Specifically, accurately determining individualized heart rate variability would require multiple assessment sessions [40], potentially increasing trial burden and complexity and leading some clinical experts to advise against its use.

Therefore, the extent to which this can be applied within the NHS setting in a clinical population needs further consideration and should be piloted in future research. Clinicians in attendance suggested that technology used in the NHS should remain simple and inexpensive, to not only facilitate staff in administering the intervention, but to also and importantly support interested patients in implementing this intervention at home or over an extended period of time.

### PPI Feedback

Following 2 online PPI group discussions in April 2023 and March 2024, collaborators provided their feedback and evaluated the PPI activities. Participants rated their satisfaction on a 10-point Likert scale, the results of which are presented in Table 3 below. As evident, attendees reported no negative feedback and highlighted improved understanding of concepts like interoception.

**Table 3.** Participants’ mean satisfaction (0-10 Likert scale) following April 2023 and March 2024 discussions.

Discussion session	Overall satisfaction, mean (SD)
April 2023	8.6 (1.67)
March 2024	9.0 (0.93)

Specifically, in response to the open-ended question, a clinical advisor noted that participating in the trial hugely increased their interest in working with interoceptive difficulties in eating disorders. Additionally, a lived experience advisory panel member was very satisfied participating in the trial, noting that it “set a new bar for lived-experience involvement. Researchers have been thoughtful, detail-oriented, and disability-literate. While my own involvement was curtailed at some points by active health problems, I always felt listened to and it was evident that researchers sought to make the process accessible, predictable, and transparent throughout.” As in previous PPI efforts, collaborators expressed their interest in the research conducted, gaining knowledge on the topics discussed, while lived-experience members felt empowered [6]. In contrast, other lived experience advisory panel collaborators considered the discussion challenging to follow, saying, “Having little or no academic or clinical experience in this field of study I felt rather ill-equipped to share my lived experience or couch them in the language that academics use.” This barrier was also observed by the research team when discussing research findings, as clinical-academic steering committee members contributed more during the discussion. To raise the level of discussion and contribute to efficient collaboration with the possibility of coproduction, training opportunities for both experts by experience and researchers conducting PPI activities should be prioritized in the planning of a research trial [31]. Moreover, researchers should explore opportunities available within the organization supporting their research, which can provide guidance throughout.

## Discussion

### Key Findings

The group discussions conducted throughout the design, delivery, and after-trial phases highlighted several key findings relevant to the feasibility, safety, and future implementation of the InMe intervention. Feedback regarding participants’ prior experience with slow-paced breathing led to the introduction of baseline assessments and the development of an adverse event protocol, ensuring appropriate responses in the instance of light-headedness, panic, or breathing discomfort. Adaptations to the stressor procedures were also informed by collaborator input, emphasizing the importance of tailoring experimental stressors to the characteristics of the university student population. Notably, all changes proposed by lived experience experts were successfully implemented during the trial design phase. It is important to acknowledge, however, that the scope of their input was limited by practical considerations, including study funding and timelines, which may limit the feasibility of some suggestions in other contexts. Collectively, these findings underscored the value of co-design in enhancing participant safety and methodological robustness.

Further discussions focused on the clinical translation and future optimization of the intervention. The differing recommendations from researchers and experts by experience regarding the future implementation of the trial indicate that further discussion and piloting with clinicians is needed to determine the most appropriate mode of delivery. It emerged that the InMe trial should be delivered alongside established standardized treatment approaches with a health care professional, rather than as a standalone or online intervention, due to concerns regarding motivation and anxiety in ED populations.

The intervention demonstrated symptom-specific benefits for individuals with EDs but not for somatic symptom disorders, suggesting that different maintaining mechanisms cannot be generalized. Withdrawal rates in individuals with subclinical EDs and somatic symptom disorders highlighted challenges in adherence, reinforcing recommendations for graded exposure to biofeedback and the use of diagnosis-specific stressors in future studies. Finally, while tailoring the breathing rhythm to individual physiology may facilitate stress reduction, concerns regarding technical complexity and scalability suggest that any future clinical application, particularly within health care settings, should prioritize simplicity, affordability, and ease of implementation.

### Limitations

After the design phase, recruitment, participant testing, data analysis, and discussion proceeded without the continued involvement of experts by experience, representing a key limitation of the study. Furthermore, collaborators were not included as coauthors on the manuscript, despite growing recognition that coauthorship constitutes good practice within PPI research [41]. The impact log also reflects that clinical-academic steering committee members contributed more than lived experience advisory panel members, which is commonly observed when online meetings and conversation dynamics are not appropriately managed [33]. One way to address this could be by holding online discussion groups or in-person workshops exclusively with lived experience advisory panel members to align the discussion with patients’ needs, creating a less intimidating environment, while educating members of the public on the specific research discipline [42]. Alternatively, when resources permit, longer workshops with specific prompts could be used to address power dynamics, and training could be provided to PPI partners. Such training has been shown to be a key driver of efficient collaboration [43]. Additionally, lived experience advisory panel members were outnumbered by clinical-academic steering committee members, primarily due to the lack of financial reimbursement and the reliance on social media and previous contacts for recruitment. One of the main barriers to efficient collaboration is the issue of limited funding for PPI activities, which has been highlighted in previous research as a persistent challenge [44,45] and should be carefully addressed in future research proposals. Many organizations supporting the research trial could also offer access to databases of lived experience experts who have previously partnered in research, such as the recently formed Center for Equality Research in Brain Sciences at University College London. Moreover, due to the lack of involvement of community members in the PPI activities, the panels consisted of more health care professionals with a different primary source of income. Unequal dynamics between health care professionals and experts by experience during PPI activities should be particularly monitored, as it can hinder meaningful change [46,47]. Thus, funding opportunities should consider monetary compensation, which is equivalent to a full-time source of income, for members of the public to be involved in research.

### Future Considerations

#### Coresearch

Future research should explore opportunities to collaborate with coresearchers in conducting research and disseminating findings, as this enables meaningful involvement and prioritizes patient-identified priorities [48]. This approach aligns with NIHR guidance and supports lived experience advisory panel members in developing a sense of ownership, empowerment, and shared responsibility in decision-making throughout the research process [41].

#### Funding

Funders of subclinical, feasibility, and pilot studies should consider allocating budgets for PPI activities. Effective PPI is greatly beneficial but requires time and appropriate preparation and funding [49].

#### Community Representation

Due to the lack of involvement of community members in the PPI activities, panels commonly consist of more health care professionals with a different primary source of income. Unequal dynamics between health care professionals and experts by experience during PPI activities should be particularly monitored, as it can hinder meaningful change [46,47]. Many organizations supporting the research trial could also offer access to databases of lived experience experts who have previously partnered in research, such as the recently formed Center for Equality Research in Brain Sciences in the United Kingdom.

#### Training and Tools

Further time should be invested in planning the goals and intentions of the aforementioned training activities as part of a general onboarding process for the trial team, as early engagement with collaborators was identified as the main facilitator in refining the research protocol and procedures [50]. Previous reviews on the topic have identified the need for substantial training and time for experts by experience, clinicians, and academics to develop good rapport with each other and efficient communication channels, in order to identify common research priorities [6,33,49,51]. Additionally, initiatives could incorporate training tools that could facilitate and support the experts by experience and members of the public involvement team [52]. For instance, this could include interactive training courses provided by the NIHR [53], along with guidance for researchers conducting PPI activities. This guidance may include the 4Pi framework, developed by the National Involvement Partnership project [54], which outlines principles for good practice, as well as for monitoring and evaluating PPI, developed by experts by experience. Similarly, the UK Standards for Public Involvement, a partnership initiative across the 4 nations since 2016, aims to improve the quality of PPI in research [31].

### Conclusion

Overall, the findings from this study highlight the importance of meaningful collaboration with health care professionals and individuals with lived experience across the development of the intervention, trial design, and implementation. PPI contributors informed and collaborated in the design, delivery, future planning, and interpretation of results for the InMe trial, including the development of trial materials, refinement of participant communications, input into the nature of the intervention, contributions to the statistical analysis plan, and support with dissemination of findings. The intervention was deemed most appropriate as an adjunct to standard care, particularly as a prehabilitation tool for individuals on waiting lists. Discussions with PPI contributors on how participants can better engage with slow breathing suggested that future studies may benefit from adjusting breathing rhythms to individual participants to support more efficient downregulation of heart rate following stressor exposure. Another important contribution to future research design was the recommendation to develop a graded therapeutic intervention to ease participants into the process, whereby cardiac biofeedback, interoceptive belief training, and stressor exposure are introduced progressively across multiple sessions. Challenges related to engagement and adherence, particularly among outpatient populations, further reinforce the need for such graded approaches, alongside diagnosis-specific procedures and simple delivery models. PPI members expressed overall satisfaction with the PPI activities and reported increased interest in interoception research. However, the research team acknowledges that there is opportunity for improvement, emphasizing the importance of early planning of PPI activities to enable impactful coproduction with members of the public and experts by experience, which requires appropriate funding. Finally, this study highlights the ongoing underrepresentation of minorities in research, which does not reflect the diversity of the setting in which this work was conducted and should be actively addressed in future studies.

## Abbreviations

ED: eating disorder
InMe: Interoceptive Insight and Metacognitive Efficacy beliefs
NHS: National Health Service
NIHR: National Institute for Health and Care Research
PPI: patient and public involvement
RCT: randomized controlled trial
SOP: standardized operating procedure
TSST: Trier Social Stress Test
